# Cardio-Vasculo-Renal Benefits of SGLT2 Inhibitors in Heart Failure: A Retrospective Study from a Lower-Resource Tertiary Center

**DOI:** 10.3390/medicina62020256

**Published:** 2026-01-26

**Authors:** Olivia-Maria Bodea, Gabriel Florin Răzvan Mogoș, Nilima Rajpal Kundnani, Abhinav Sharma, Ovidiu Adam, Daniel Marius Duda-Seiman, Dana Velimirovici, Marioara Nicula-Neagu, Ovidiu Horea Bedreag, Simona Dragan

**Affiliations:** 1 Doctoral School, “Victor Babes” University of Medicine and Pharmacy, 3000041 Timisoara, Romania; 2 University Clinic of Internal Medicine and Ambulatory Care, Prevention and Cardiovascular Recovery, Department VI-Cardiology, “Victor Babes” University of Medicine and Pharmacy, 3000041 Timisoara, Romaniadana.velimirovici@umft.ro (D.V.);; 3 Department of Surgery, University of Medicine and Pharmacy of Craiova, 200349 Craiova, Romania; 4 Research Centre of Timisoara, Institute of Cardiovascular Diseases, “Victor Babes” University of Medicine and Pharmacy, 3000041 Timisoara, Romania; 5 Department of Pediatric Surgery and Orthopedics, “Victor Babes” University of Medicine and Pharmacy, Eftimie Murgu Square, No. 2, 300041 Timisoara, Romania; 6 Physiology Discipline, Faculty of Bioengineering of Animal Resources, University of Life Sciences “King Mihai I” from Timisoara, 300645 Timisoara, Romania; mnicula@animalsci-tm.ro; 7 Clinic of Anaesthesia and Intensive Care, Emergency County Hospital “Pius Brinzeu”, 300736 Timisoara, Romania; 8 Faculty of Medicine, “Victor Babes” University of Medicine and Pharmacy, 300041 Timisoara, Romania

**Keywords:** SGLT2i, eGFR, kidney function, treatment algorithms, glycemic control

## Abstract

*Background and Objectives:* Heart failure frequently coexists with CKD, compounding prognosis via cardio-renal interplay. Sodium glucose cotransporter 2 (SGLT2) inhibitors have demonstrated cardiovascular and renal benefits in randomized trials, but data remain limited in real-world lower-resource settings. *Materials and Methods:* We conducted a retrospective single-center cohort study at a tertiary university hospital in western Romania, including adults with chronic HF and LVEF ≤ 45%, monitored between 2021–2024. Patients were stratified based on receipt of SGLT2 inhibitors. The primary endpoint was a composite of cardiovascular death, HF hospitalization, or ≥40% sustained decline in eGFR/initiation of KRT. Annual eGFR slope was analyzed to assess renal trajectory. *Results:* Among 240 patients, treatment with SGLT2 inhibitors was associated with a lower risk of the composite cardio-vasculo-renal endpoint compared with no treatment (adjusted HR 0.70, 95% CI 0.50–0.98). The reduction was primarily driven by fewer heart failure hospitalizations. Decline in kidney function was slower among SGLT2 inhibitor-treated patients in longitudinal mixed-effects analyses. *Conclusions:* In this retrospective cohort, SGLT2 inhibitor use was associated with fewer cardio-renal events and a slower decline in kidney function. Given the observational design and residual confounding risk, these findings should be considered hypothesis-generating but provide implementation-relevant signals supporting further prospective evaluation.

## 1. Introduction

Heart failure (HF) is a leading cause of morbidity and mortality worldwide and carries a lifetime risk of around 20–25% in the general population, with persistently high rates of hospitalization and reduced survival despite advances in therapy [1]. Large contemporary registries and guideline documents emphasize that HF is frequently accompanied by multiple comorbidities, particularly chronic kidney disease (CKD), which further worsens prognosis and limits the use of guideline-directed medical therapy [2].

CKD is present in up to half of patients with chronic HF and is independently associated with higher all-cause and cardiovascular mortality, a greater risk of HF hospitalizations, and a faster decline in kidney function. HF and CKD often coexist within a complex bidirectional interaction, in which reduced cardiac output, neurohormonal activation, venous congestion, and systemic inflammation contribute to progressive loss of renal function, while impaired kidney function aggravates volume overload, hypertension, and vascular stiffness [2,3]. This interdependence has led to the concepts of “cardio-renal” and broader “cardio-vasculo-renal” syndromes, underlining the need for therapies that provide integrated protection across the heart, vasculature, and kidneys [4,5].

Originally developed as glucose-lowering agents, Sodium glucose cotransporter 2 (SGLT2) inhibitors rapidly emerged as cardio-renal protective therapies. Across randomized trials in HFrEF and across the EF spectrum, SGLT2 inhibitors consistently reduce heart failure hospitalization and improve renal outcomes, with benefits observed irrespective of diabetes status. This diabetes-independent protection supports their role as foundational therapy targeting the integrated cardio-renal axis rather than glycemic control alone [6,7,8,9,10,11,12].

The mechanisms underpinning these cardio-vasculo-renal benefits are multifactorial and extend beyond glucose-lowering. Experimental and clinical data suggest that SGLT2 inhibitors induce a modest osmotic diuresis and natriuresis, reduce plasma and interstitial volumes, and lower blood pressure and arterial stiffness, thereby improving ventricular loading conditions without significant neurohormonal activation [7,13,14]. At the renal level, restoration of tubulo-glomerular feedback reduces intraglomerular pressure and albuminuria, slowing structural damage to the glomerulus. Additionally, SGLT2 inhibition may favorably influence myocardial and vascular metabolism, promote a shift toward more efficient fuel utilization, reduce uric acid, and exert anti-inflammatory and antifibrotic effects within the heart and kidney [15,16,17].

On the basis of this evidence, contemporary HF guidelines from the European Society of Cardiology (ESC) now recommend SGLT2 inhibitors as one of the four pillars of foundational therapy in patients with HFrEF, alongside renin–angiotensin system inhibition (preferably with angiotensin receptor–neprilysin inhibitors), beta-blockers, and mineralocorticoid receptor antagonists [1].

Similarly, guidelines for diabetes and CKD, such as the KDIGO 2022 Clinical Practice Guideline and related consensus statements, endorse SGLT2 inhibitors as first-line therapy in patients with type 2 diabetes and CKD, particularly those with albuminuria, because of their proven kidney and cardiovascular protection [18].

Despite the robust trial and guideline evidence, it remains important to understand how these benefits translate into routine clinical practice, where patients often present with advanced multimorbidity, varying degrees of renal impairment, and heterogeneous background therapy. Many randomized trials were not specifically designed to evaluate integrated cardio-vasculo-renal endpoints that simultaneously capture cardiovascular death, HF hospitalization, and major renal events in real-world HF cohorts. Recent observational studies and meta-analyses suggest that SGLT2 inhibitor therapy is associated with fewer HF hospitalizations, improved survival, and slower CKD progression in clinical practice, but data focusing on composite cardio-vasculo-renal outcomes in unselected HF populations remain limited [19,20].

In this context, we designed a single-center retrospective cohort study of patients with chronic HF and reduced or mildly reduced ejection fraction, managed in a tertiary clinic in western Romania.

In spite of the aforementioned evidence and guideline driven therapy, Romania represents a lower-to-middle resource healthcare context within the European Union. Here implementation of advanced heart failure therapies often lags behind Western European adoption due to economic constraints, medication accessibility, and structural healthcare disparities. In contrast to nations with established, protocol-driven heart failure management frameworks, Romanian cardiology practice frequently requires prioritization of therapies based on affordability and availability rather than solely on clinical efficacy [21,22,23]. This makes the cardiac population in Romania particularly valuable for studying real-world therapeutic performance of SGLT2 inhibitors in settings where prescription is not automatic or guideline-driven, but rather selectively applied. By analyzing outcomes in such an environment, our study provides insight into the cardio-renal effects of SGLT2 inhibition under pragmatic prescribing conditions that more closely resemble healthcare realities in resource-limited regions both within Eastern Europe and globally.

Although SGLT2 inhibitors are formally reimbursed in Romania for eligible heart failure phenotypes, real-world access and implementation remain heterogeneous. Prescription frequently depends on institutional availability, outpatient follow-up continuity, physician familiarity with cardio-renal indications, and local administrative pathways rather than automatic guideline-driven uptake. Thus, the term “lower-resource setting” in this study refers not to the absence of reimbursement, but to structural and organizational constraints affecting real-world implementation.

The aim of the present analysis was to evaluate the association between SGLT2 inhibitor use and a composite cardio-vasculo-renal endpoint that included cardiovascular death, HF hospitalization, and major renal events (≥40% decline in estimated glomerular filtration rate or initiation of kidney replacement therapy), and to assess the impact of SGLT2 inhibitors on the rate of eGFR decline during follow-up. All this is being carried out in a lower settings tertiary center in western Romania. Secondarily, we aim to provide real-world implementation evidence capable of supporting earlier and broader adoption of SGLT2 inhibition in routine heart failure care within a specific healthcare setting. We hypothesized that treatment with SGLT2 inhibitors, on top of guideline-directed medical therapy, would be associated with a lower incidence of the composite endpoint and a slower decline in kidney function compared with patients not receiving SGLT2 inhibitors.

## 2. Materials and Methods

### 2.1. Study Design and Setting

This is a retrospective observational cohort study conducted in the Institute of Cardiovascular Diseases, Timisoara, a tertiary university clinical center. The analysis included adult patients with chronic heart failure who were followed between January 2021 and December 2024. Clinical, laboratory, demographic, and pharmacologic data were extracted from institutional electronic patient records and manually cross-validated by two independent investigators to ensure accuracy. The study adhered to the ethical principles of the Declaration of Helsinki [24] and was conducted in full compliance with the General Data Protection Regulation, GDPR [25], regarding anonymization, confidentiality, and secure data handling.

### 2.2. Ethical Approval

Ethical approval for this study was granted by the Review Board of the University of Medicine and Pharmacy of Timisoara affiliated Institute of Cardiovascular Diseases (Approval number: 2099/16 March 2022). All patients signed a standard institutional consent form at the time of admission, which authorized the storage and research use in future of anonymized clinical data for scientific purposes. All data used in analysis were fully anonymized prior to processing, and no identifiable patient information was included in the dataset.

The study was approved as a retrospective observational project, authorizing access to anonymized clinical records. Due to reduced hospitalizations during the COVID-19 pandemic, retrospective data extraction was continued under the same approval to include patients hospitalized up to December 2024; no data were accessed prior to approval, no patient contact occurred, and all data were fully anonymized.

Prescription of SGLT2 inhibitors was performed exclusively at the independent discretion of the treating physicians, prior to and fully independent of this retrospective analysis, and the study did not involve any form of therapeutic intervention, protocol-driven treatment modification, or influence on clinical decision-making. The purpose of the present study was strictly observational and hypothesis-generating in nature, aiming to analyze real-world outcomes and provide implementation-relevant evidence without directing or altering routine clinical practice.

Patients listed as having “refused consent” in the flow diagram refer to refusal of secondary data use authorization; all patients provided standard clinical consent at admission. Only anonymized data from patients permitting research reuse were included in the analysis.

### 2.3. Study Population

The study included adult patients aged 18 years or older with a documented diagnosis of chronic heart failure. Patients were eligible if they had left ventricular ejection fraction (LVEF) ≤ 45%, NYHA functional class II–IV, at least two estimated glomerular filtration rate measurements (eGFR, mL/min/1.73 m^2^) during follow-up, and a minimum follow-up duration of 12 months. Patients were excluded if they had type 1 diabetes mellitus, acute kidney injury within the 30 days prior to baseline, history of kidney transplantation, dialysis at baseline, active malignancy, advanced hepatic dysfunction, systemic infection, or missing core baseline variables such as LVEF, eGFR, or SGLT2 inhibitor status.

### 2.4. Exposure Definition and Index Date

To avoid immortal time and index-date bias, cohort entry (“baseline”) was defined uniformly for all patients as the first qualifying outpatient or inpatient heart failure assessment during the study period at which all inclusion criteria were met.

Exposure to sodium–glucose cotransporter-2 inhibitors (SGLT2i) was modeled as a time-dependent variable. All patients contributed unexposed person-time from baseline until initiation of SGLT2i therapy. Following initiation, patients contributed exposed person-time irrespective of subsequent treatment discontinuation (intention-to-treat approach).

Patients who never initiated SGLT2i therapy remained unexposed throughout follow-up. This time-dependent exposure framework was selected a priori to prevent immortal time bias and ensure valid time-to-event comparisons.

### 2.5. Outcomes

The primary endpoint was a cardio-vasculo-renal composite consisting of the first occurrence of cardiovascular death, first hospitalization for heart failure, or a major renal event defined as a ≥40% sustained decline in eGFR (mL/min/1.73 m^2^) or initiation of kidney replacement therapy (hemodialysis or peritoneal dialysis). Secondary outcomes included each individual component of the composite endpoint, all-cause mortality, myocardial infarction, ischemic stroke, total number of heart failure hospitalizations per patient, and the annualized eGFR slope (mL/min/1.73 m^2^ per year) during follow-up.

### 2.6. Statistical Analysis

All analyses were performed using IBM SPSS Statistics version 26 and R version 4.3.3. Data distribution was assessed using the Shapiro–Wilk test. Normally distributed variables were analyzed using Student’s *t*-test, and non-normally distributed variables using the Mann–Whitney U test. Categorical variables were compared using the chi-square or Fisher’s exact test. Kaplan–Meier curves were constructed for the primary composite endpoint and compared using the log-rank test.

Time-to-event outcomes were analyzed using Cox proportional hazards models with time-dependent SGLT2i exposure. Multivariable models adjusted for age, sex, baseline eGFR, diabetes status, NYHA class, ischemic etiology, atrial fibrillation, systolic blood pressure, anemia, and use of guideline-directed medical therapy (RAAS inhibitors or ARNI, beta-blockers, and mineralocorticoid receptor antagonists).

To further address confounding by indication, a propensity score overlap-weighting approach was applied as the primary analysis. Propensity scores were estimated using logistic regression incorporating the same covariates. Covariate balance was assessed using standardized mean differences (SMD), with values < 0.10 considered acceptable.

Covariate balance before and after overlap weighting is shown in Supplementary Table S1.

Traditional multivariable Cox models were retained as sensitivity analyses.

Proportional hazards assumptions were verified using Schoenfeld residuals. The annualized eGFR slope (mL/min/1.73 m^2^ per year) was calculated using linear mixed-effects modeling. A *p*-value < 0.05 was considered statistically significant.

To address potential confounding by indication inherent to the retrospective design, multivariable Cox proportional hazards models were adjusted for clinically relevant covariates known to influence both treatment allocation and outcomes, including age, sex, baseline eGFR, diabetes status, NYHA functional class, ischemic etiology, atrial fibrillation, and background guideline-directed medical therapy.

### 2.7. Outcome Definitions and Ascertainment

Heart failure hospitalization was defined as an unplanned hospital admission lasting ≥24 h with a primary diagnosis of heart failure requiring intravenous diuretic therapy.

The renal component of the composite endpoint was defined as a ≥40% sustained decline in eGFR from baseline, confirmed by at least two measurements obtained ≥30 days apart. eGFR values obtained within the first 30 days after SGLT2i initiation were excluded from renal endpoint assessment to account for the expected acute hemodynamic dip. Initiation of kidney replacement therapy (hemodialysis or peritoneal dialysis) was also considered a renal event.

Myocardial infarction and ischemic stroke were identified based on discharge diagnoses and confirmed by structured chart review. All outcome events were independently verified by two investigators blinded to treatment exposure.

## 3. Results

### 3.1. Baseline Characteristics

A total of 460 patients with chronic heart failure and LVEF ≤ 45% were initially screened for eligibility during the study period. After systematic exclusion, the final cohort was represented by 240 patients. As presented before, a number of demographic variables were acquired and compared between groups. This is illustrated in Table 1.

The two groups were comparable in age and sex distribution, with similar heart failure severity reflected by NYHA functional class and LVEF. Comorbidity burden was also balanced, including prevalence of CKD, type 2 diabetes mellitus, atrial fibrillation, ischemic etiology, and prior myocardial infarction. Background guideline-directed medical therapy was broadly comparable between groups, including RAAS inhibition, beta-blockers, mineralocorticoid receptor antagonists, loop diuretics, and statins, supporting interpretability of outcome comparisons.

**Figure 1:**
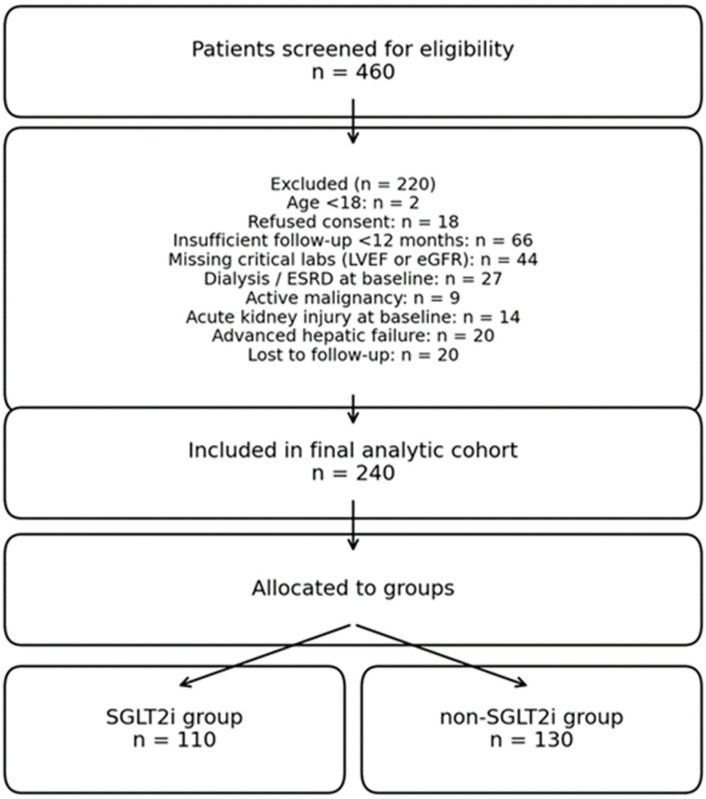
Study flow diagram showing patient screening, exclusions, and final analytic cohort.

As shown in Figure 1, after sequential exclusion based on age, consent status, comorbid renal and hepatic conditions, insufficient follow-up, missing analytical variables, refusal to participate, and loss to follow-up, the final analytic cohort comprised 240 patients. Figure 1 depicts the narrowing of the cohort, with explicit exclusion reasons and sample sizes indicated in each step, resulting in two well-defined analysis groups.

Of the 240 included patients, 110 (45.8%) received an SGLT2 inhibitor while 130 (54.2%) remained untreated.

Baseline characteristics were comparable across the two groups (SGLT2I vs. non-SGLT2I) with respect to age (67.4 ± 9.3 vs. 68.2 ± 9.7 years), sex, degree of systolic dysfunction (LVEF 31.8 ± 6.2% vs. 31.1 ± 6.5%), baseline kidney function (eGFR 47.9 ± 15.8 vs. 46.8 ± 16.4 mL/min/1.73 m^2^), and NYHA functional class distribution. Median follow-up was 24 months (IQR 18–30) in both arms. The median duration of SGLT2 inhibitor exposure was 22 months (IQR 17–28).

### 3.2. Primary Outcome

During follow-up, the primary cardio-vasculo-renal composite endpoint occurred in 27/110 (24.1%) SGLT2I-treated patients and 45/130 (35.0%) untreated patients (*p* = 0.006). The comprehensive primary and secondary outcomes are highlighted in Table 2.

In multivariable Cox regression, SGLT2i exposure was associated with a lower hazard of the primary composite endpoint, driven primarily by reduced HF hospitalization and fewer renal events (Table 2). Associations with cardiovascular and all-cause mortality did not reach statistical significance and should be interpreted as hypothesis-generating.

**Figure 2:**
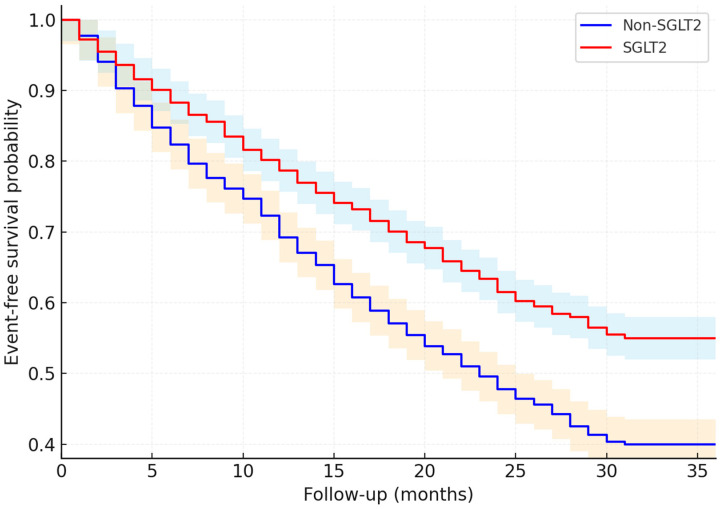
Kaplan–Meier curves for the primary composite endpoint (36 months). The primary composite endpoint was defined as first occurrence of cardiovascular death, heart failure hospitalization, or a ≥40% sustained decline in eGFR. SGLT2I: sodium–glucose cotransporter-2 inhibitor. Shaded confidence intervals reflect 95% uncertainty around survival estimates. Red = SGLT2I; Blue = non-SGLT2I; vertical ticks = censored observations. Time zero corresponds to cohort entry, defined as the first qualifying heart failure assessment during the study period. SGLT2I exposure was modeled as a time-dependent variable in adjusted analyses.

Kaplan–Meier analysis (Figure 2) showed a significantly lower cumulative incidence of the primary composite endpoint in the SGLT2i group compared with the non-SGLT2i group over follow-up (log-rank *p* = 0.041).

In sensitivity analyses, the association between SGLT2i use and the primary composite endpoint remained consistent after additional adjustment for atrial fibrillation, anemia, systolic blood pressure, baseline eGFR category (<60 vs. ≥60 mL/min/1.73 m^2^), and use of mineralocorticoid receptor antagonists. No single covariate materially altered the direction or magnitude of the association.

### 3.3. Secondary Outcomes

Among individual components of the composite endpoint, heart failure hospitalization occurred less frequently in patients treated with SGLT2 inhibitors, while differences in cardiovascular and all-cause mortality did not reach statistical significance. After multivariable adjustment, the association with major renal decline was attenuated and did not remain statistically significant (Table 2).

Safety and tolerability outcomes are summarized in Supplementary Table S2. Rates of acute kidney injury, volume depletion, and diabetic ketoacidosis were low and comparable between groups, while genital mycotic infections were more frequent among SGLT2i-treated patients.

### 3.4. Renal Outcomes and eGFR Slope Analysis

Renal trajectory was assessed using linear mixed-effects models with random intercepts and slopes, incorporating all available eGFR measurements during follow-up. The CKD-EPI 2021 equation was used for eGFR estimation. The median number of eGFR measurements per patient was 6 (IQR 4–8), with a median interval of 4.2 months between assessments.

**Figure 3:**
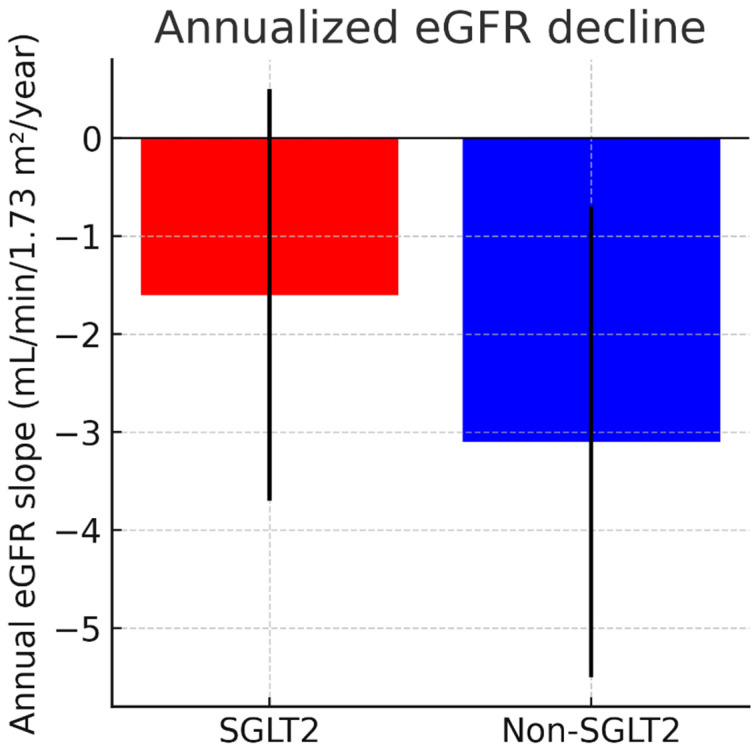
Annualized eGFR slope demonstrating slower renal decline in SGLT2I-treated patients. eGFR = estimated glomerular filtration rate; negative values indicate decline.

To distinguish acute hemodynamic effects from chronic renal preservation, a sensitivity analysis excluding eGFR values obtained within the first 30 days after SGLT2i initiation was performed, yielding consistent results (Figure 3).

Results were consistent in sensitivity analyses excluding eGFR values obtained within the first 30 days after SGLT2i initiation (Figure S1).

**Figure 4:**
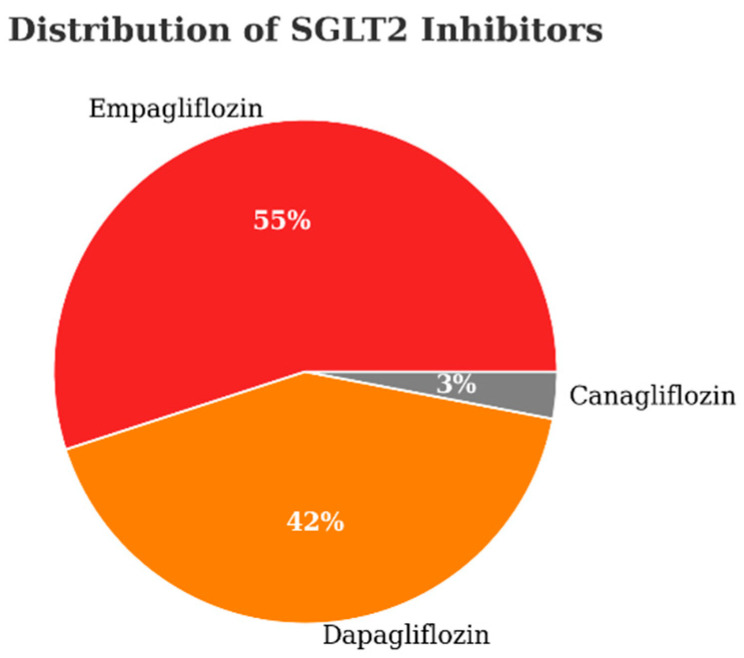
Proportions of SGLT2 inhibitors used in the treated cohort. SGLT2I: sodium–glucose cotransporter-2 inhibitor. Distribution reflects agent prescribed at baseline. Percentages are relative to the SGLT2I-treated cohort (*n* = 110) rather than the total study population (*n* = 240).

Within the SGLT2I group, Figure 4 displays the distribution of drugs used. Here, the predominant therapy was empagliflozin (55%), followed by dapagliflozin (42%), and canagliflozin (3%). The figure visually conveys the proportional use of each SGLT2I agent in the cohort, facilitating comparison of real-world prescribing patterns.

## 4. Discussion

This study adds pragmatic, implementation-relevant evidence to the randomized trial literature by evaluating an integrated cardio-renal composite endpoint and eGFR trajectory in a real-world tertiary cohort. While RCTs established class efficacy under protocolized conditions, observational data remain essential to quantify effectiveness in routine care, where multimorbidity, delayed initiation, and structural barriers can attenuate guideline uptake. By demonstrating a lower risk of cardio-renal events and slower kidney function decline in a setting where implementation is not automatic, our findings reinforce the clinical concept that SGLT2i act as disease-modifying therapies across organ systems and support earlier adoption within real-world heart failure pathways.

### 4.1. Cardiovascular Implications

The cardiovascular benefit observed in our cohort—particularly the reduction in HF hospitalization and composite adverse outcomes—reinforces the growing international consensus that SGLT2 inhibitors have become an essential component of HFrEF management. In our analysis, the event curves separated early and continued to diverge over the study period, suggesting a sustained therapeutic effect. This observation parallels the results of large randomized controlled trials, including DAPA-HF [8,10], EMPEROR-Reduced [11], which demonstrated reductions in HF exacerbations and improvements in overall clinical stability.

One particularly important clinical observation is that the beneficial effects of SGLT2 inhibitors persist across a broad range of HF severity. In EMPEROR-Reduced, even patients with markedly reduced LVEF (<30%) experienced benefit, suggesting that SGLT2 inhibition exerts cardioprotective effects even in advanced myocardial dysfunction. This aligns with accumulating evidence that these agents influence ventricular loading conditions, myocardial metabolism, and possibly intracellular ion handling in ways that extend beyond simple diuretic-like effects [26,27,28].

Moreover, the consistency of effect across diabetic and non-diabetic subgroups observed both in prior randomized trials and in our real-world cohort strengthens the argument that SGLT2I cardioprotection is not primarily glucose-mediated [13,26,28,29]. In a large meta-analysis, it has been shown that reductions in HF hospitalization were statistically indistinguishable between diabetic and normoglycemic patients receiving SGLT2I therapy [30].

While we did not detect a significant reduction in myocardial infarction or stroke, this is neither unexpected nor inconsistent with existing literature. Prior analyses, including the CANVAS and DECLARE-TIMI 58 trials, revealed that the strongest cardiovascular effects of SGLT2 inhibitors occur in heart failure and arrhythmic risk reduction rather than in atherosclerotic event suppression [7,30,31]. Taken together, our findings support the interpretation that SGLT2 inhibitors function as HF disease modifiers, not generalized anti-atherothrombotic agents.

Although cardiovascular and all-cause mortality numerically favored the SGLT2i group, these differences did not reach statistical significance and should be interpreted as hypothesis-generating rather than confirmatory.

### 4.2. Renal Implications

The attenuated renal decline in SGLT2i-treated patients in our study is concordant with major renal outcome trials, including EMPA-REG OUTCOME and DAPA-CKD, which collectively established that these medications slow CKD progression, reduce acute kidney injury risk, and preserve long-term renal function [9,12].

In our cohort, annualized eGFR loss was reduced by approximately 50%, which is clinically meaningful, especially in HF patients, where renal deterioration often accelerates treatment limitations and decompensation risk. Patients with cardiorenal syndrome frequently encounter escalating diuretic resistance, progressive azotemia, and declining tolerance to ACE-I/ARB/ARNI therapy. By mitigating renal decline, SGLT2i inhibitors may expand pharmacologic treatment windows and prevent treatment discontinuation.

Importantly, SGLT2i inhibitors appear to operate through renal mechanisms independent of glycemic status. DAPA-CKD demonstrated significant reduction in renal endpoints even in participants without diabetes [12], underscoring their broader applicability. Proposed mechanisms include improved glomerular hemodynamics through afferent arteriolar vasoconstriction, restoration of tubuloglomerular feedback, and attenuation of intrarenal hyperfiltration stress [16,32,33,34].

In a pooled kidney-outcome meta-analysis including over 47,000 patients treated with SGLT2 inhibitors, the authors concluded that these agents lowered the risk of sustained eGFR decline and kidney failure, regardless of underlying CKD etiology [35]. This supports interpretation of our findings not as incidental observations but as manifestations of a robust and reproducible renoprotective pharmacologic effect.

### 4.3. Integration with Current Clinical Practice

Current HF guidelines have rapidly evolved to incorporate SGLT2 inhibitors as key disease-modifying therapy. The ESC 2021 HF guidelines [1] and the 2022 ACC/AHA/HFSA HF guidelines [36] both designate SGLT2 inhibitors as Class I recommended therapy for patients with HFrEF, regardless of diabetes.

However, real-world implementation often lags behind guideline recommendation. Clinicians may exhibit hesitancy due to unfamiliarity, perceived concerns about volume depletion, or uncertainty regarding renal safety. Registry data from European HF cohorts show that SGLT2 inhibitors remain underused relative to guideline recommendations, with fewer than 40% of eligible HFrEF patients receiving these agents in contemporary practice [37]. A global review of real-world data concluded that SGLT2 inhibitors are still prescribed in only a minority of eligible patients with T2DM and HF/CKD, despite strong evidence and guideline endorsement [38]. In a Korean CKD cohort, SGLT2 inhibitors were substantially underused among eligible patients, with nephrologists and diabetologists citing uncertainty about renal safety, limited familiarity, and concerns about volume depletion as barriers to initiation [39]. Qualitative work in Hong Kong primary care showed that limited awareness of cardio-renal indications and lingering safety concerns contribute to slower uptake of SGLT2 inhibitors in routine practice [40]. Survey data confirm that clinicians’ hesitation is often driven by perceived safety issues—particularly volume depletion, AKI, DKA and genitourinary infections—even though these adverse events are usually manageable and serious complications are uncommon [41]. Large pharmaco-epidemiologic studies highlight marked socioeconomic gradients in SGLT2I uptake: patients with lower income or living in lower-resource settings are less likely to receive SGLT2 inhibitors, despite guideline eligibility [42]. Our findings provide reassuring empirical data demonstrating that, in a real-world tertiary care setting, in a lower-resource setting, SGLT2I treatment is associated with reliable cardio-renal benefit. In this context, “lower-resource” reflects implementation barriers rather than drug unavailability, including delayed initiation, selective prescribing, and variability in multidisciplinary heart failure care.

Additionally, the modest difference in baseline diabetic status in our cohort (57.3% vs. 51.9%) did not materially influence observed outcomes, underscoring the glucose-independent therapeutic rationale. HF clinicians should thus regard SGLT2 inhibitors as intrinsic HF therapeutics rather than as antihyperglycemic agents applied opportunistically when diabetes happens to coexist.

Our data also reinforce the concept of early initiation. The event-curve separation was visible by mid-follow-up and widened thereafter. Therefore, early adoption following HFrEF diagnosis—alongside β-blocker, RAAS inhibition or ARNI, and MRA therapy—may be ideal.

### 4.4. Translational and Guideline-Implementation Relevance

Although SGLT2 inhibitors are recommended as Class I therapy for HFrEF, real-world uptake remains substantially below guideline expectations [36], particularly in lower-resource healthcare systems. Data from the Swedish Heart Failure Registry show that fewer than half of eligible patients receive SGLT2 inhibitors in routine practice [37]. Implementation barriers include clinician safety concerns, limited cardio-renal integration, and unfamiliarity with the class despite strong safety data [40,41]. Access and affordability further limit adoption in low- and middle-income regions [22,23], while socioeconomic disparities significantly influence initiation even within high-income health systems [42].

This study provides implementation-relevant, hypothesis-generating evidence from a real-world tertiary care setting, highlighting the potential cardio-renal benefits of SGLT2i therapy under heterogeneous prescribing conditions. By demonstrating consistent associations with reduced heart failure hospitalization and slower renal decline, these findings provide pragmatic, hypothesis-generating implementation signals relevant to heterogeneous real-world care settings.

### 4.5. Cardiac Remodelling and Endothelial Effects

Beyond hemodynamic decongestion, emerging evidence suggests that SGLT2i may favorably influence cardiac remodeling by reducing myocardial wall stress, interstitial fibrosis, and adverse ventricular hypertrophy, potentially through improved myocardial energetics, attenuation of inflammatory signaling, and modulation of intracellular ion homeostasis. In parallel, SGLT2i appear to ameliorate endothelial dysfunction by improving nitric oxide bioavailability, reducing oxidative stress and vascular inflammation, and decreasing arterial stiffness, thereby improving vascular compliance and microvascular function in heart failure [43,44].

Emerging evidence further suggests that SGLT2 inhibitors exert pleiotropic electrophysiological and myocardial effects, including modulation of autonomic tone, reduction in arrhythmic vulnerability, and improvements in cellular bioenergetics, which may contribute to their broader cardiovascular benefits beyond hemodynamic unloading [45].

### 4.6. Study Strengths

The use of hard clinical endpoints—including cardiovascular death, HF hospitalization, and major renal impairment—enhances reliability and reduces subjective interpretation bias. Furthermore, follow-up duration of 24 months allows meaningful renal slope analysis, the stratification of exclusion reasons enhances transparency, the censoring events were clearly documented and consistent pharmacologic background therapies allowed cleaner comparisons

Unlike some smaller single-center retrospective studies, the present dataset includes a broad spectrum of HF severity and CKD stage, reflecting actual case heterogeneity encountered in clinical practice.

### 4.7. Study Limitations

While the study is retrospective in design, several methodological strengths mitigate bias. The absence of randomization may introduce confounding by indication, yet baseline characteristics between groups were highly comparable. We accounted for known prognostic variables in multivariable modeling, including age, eGFR, NYHA class, and HF etiology, which strengthens internal validity.

Exclusion of patients with incomplete datasets may reduce generalizability; however, this approach prevented misclassification bias and ensured analytical integrity. Although this is a single-center study, the composition of the patient population closely mirrors real-world HF demographics in tertiary cardiology care, enhancing applicability to similar clinical environments.

Furthermore, while we did not analyze differences among individual SGLT2I agents, results from multiple major trials and pharmacologic analyses support the understanding that these agents exhibit class-consistent effects [46,47,48], making differentiation at drug-specific level less clinically imperative.

As with all retrospective observational studies, residual confounding and confounding by indication cannot be fully excluded. Treatment allocation was influenced by clinical judgment and local practice patterns rather than randomization. Although extensive multivariable adjustment and sensitivity analyses were performed, unmeasured factors may still contribute to the observed associations. Therefore, causal inference should be avoided, and the findings should be interpreted as hypothesis-generating.

Although time-dependent exposure modeling was used to mitigate immortal time bias, such approaches rely on correct specification of treatment timing and assume no unmeasured time-varying confounders.

### 4.8. Future Directions

Future efforts may include prospective observational expansion or randomized pragmatic trial designs examining echocardiographic evolution, natriuretic peptide trends, and functional testing under SGLT2I therapy. Analysis of right ventricular function, pulmonary pressures, and long-term arrhythmic outcomes may also provide further insight.

Additionally, exploration of SGLT2I effects across HF phenotypes beyond HFrEF—including HFpEF and HF with mildly reduced EF—is of increasing importance given recent evidence of benefit in these populations as well [40,49,50].

Finally, investigation into patient adherence, tolerability, and real-world safety monitoring will be vital to integrating SGLT2I inhibition seamlessly into chronic HF care workflows—real-world data already suggest that SGLT2I inhibitors are generally well tolerated, with manageable adverse-event profiles, acceptable discontinuation rates, and no unexpected increase in acute kidney injury, even among elderly or frail patients [51,52,53,54].

## 5. Conclusions

In this retrospective cohort study, treatment with SGLT2 inhibitors was associated with a lower incidence of cardio-renal adverse events and a slower decline in kidney function over two years of follow-up. Given the observational design and potential for residual confounding, these findings should be interpreted as hypothesis-generating rather than causal. Nonetheless, they support further prospective and pragmatic studies evaluating SGLT2i therapy in diverse real-world heart failure populations.

## Figures and Tables

**Table 1:** Baseline characteristics of study cohort.

Variable	Unit	SGLT2I (*n* = 110)	Non-SGLT2I (*n* = 130)	*p*-Value
Age	Years	67.4 ± 9.3	68.2 ± 9.7	0.28
Male sex	%	62.7%	61.9%	0.86
BMI	kg/m^2^	28.3 ± 4.2	28.6 ± 4.6	0.51
NYHA III–IV	%	59.1%	61.5%	0.59
LVEF	%	31.8 ± 6.2	31.1 ± 6.5	0.29
Ischemic HF etiology	%	68.6%	66.9%	0.69
Heart rate	bpm	76 ± 12	77 ± 13	0.40
SBP	mmHg	122 ± 15	123 ± 16	0.47
DBP	mmHg	73 ± 9	72 ± 9	0.31
Serum creatinine	mg/dL	1.45 ± 0.36	1.49 ± 0.41	0.18
eGFR baseline	mL/min/1.73 m^2^	47.9 ± 15.8	46.8 ± 16.4	0.49
CKD (eGFR < 60)	%	74.5%	75.0%	0.90
Type 2 Diabetes Mellitus	%	57.3%	51.9%	0.24
Atrial fibrillation	%	35.0%	36.9%	0.66
Coronary artery disease	%	72.7%	73.5%	0.86
Prior MI	%	38.6%	41.5%	0.49
Anemia	%	41.8%	43.1%	0.78
RAAS inhibitor	%	88.2%	87.0%	0.71
Beta-blocker	%	92.3%	90.7%	0.54
MRA	%	67.7%	65.4%	0.61
Loop diuretic	%	85.9%	87.3%	0.67
Statin	%	76.4%	78.5%	0.58

Values are presented as mean ± standard deviation for continuous variables and as percentages for categorical variables. SGLT2I: Sodium-glucose cotransporter-2 inhibitors; BMI: body mass index; NYHA: New York Heart Association functional class; LVEF: left ventricular ejection fraction; SBP: systolic blood pressure; DBP: diastolic blood pressure; eGFR: estimated glomerular filtration rate; CKD: chronic kidney disease; RAAS: renin–angiotensin–aldosterone system; MRA: mineralocorticoid receptor antagonist. Statistical comparisons were performed using Student’s *t*-test (continuous variables) and chi-square test (categorical variables). No significant between-group differences were found at baseline.

**Table 2:** Primary and Secondary Outcomes.

Outcome	SGLT2i (*n* = 110)	non-SGLT2i (*n* = 130)	Adjusted HR (95% CI)	*p*-Value
Primary composite	26 (23.6%)	44 (33.8%)	0.70 (0.50–0.98)	0.038
HF hospitalization	18 (16.4%)	36 (27.7%)	0.65 (0.44–0.96)	0.031
CV death	11 (10.0%)	17 (13.1%)	0.82 (0.50–1.34)	0.41
Major renal decline	13 (11.8%)	21 (16.2%)	0.78 (0.52–1.15)	0.21
All-cause mortality	14 (12.7%)	22 (16.9%)	0.80 (0.52–1.23)	0.31

HF: heart failure; CV: cardiovascular; Composite endpoint includes first occurrence of HF hospitalization, cardiovascular death, or ≥40% sustained decline in eGFR. Myocardial infarction and stroke were recorded as adjudicated secondary ischemic outcomes. Decline in renal function is expressed both as final eGFR and annualized eGFR slope. Statistical significance was defined as *p* < 0.05. Adjusted Cox models compare SGLT2i vs. non-SGLT2i. Covariates: age, sex, baseline eGFR, diabetes, NYHA class, ischemic etiology, RAAS inhibitor use (plus AF and anemia in sensitivity analysis, if reported). Time-to-first-event outcomes are summarized using multivariable Cox proportional hazards models and reported as adjusted hazard ratios (HRs) with 95% confidence intervals.

## Data Availability

The original contributions presented in this study are included in the article. Further inquiries can be directed to the corresponding author.

## Supplementary Materials

The following supporting information can be downloaded at: https://www.mdpi.com/article/10.3390/medicina62020256/s1. Table S1: Propensity score covariate balance before and after overlap weighting; Table S2: Safety and tolerability outcomes; Figure S1: Sensitivity analysis of eGFR slope excluding early hemodynamic dip.

## Abbreviations

ACEI	Angiotensin-Converting Enzyme Inhibitor
AKI	Acute Kidney Injury
ARB	Angiotensin Receptor Blocker
ARNI	Angiotensin Receptor–Neprilysin Inhibitor
BMI	Body Mass Index
CKD	Chronic Kidney Disease
CV	Cardiovascular
DBP	Diastolic Blood Pressure
DKA	Diabetic Ketoacidosis
eGFR	Estimated Glomerular Filtration Rate
ESC	European Society of Cardiology
HF	Heart Failure
HFpEF	Heart Failure with Preserved Ejection Fraction
HFmrEF	Heart Failure with Mildly Reduced Ejection Fraction
HFrEF	Heart Failure with Reduced Ejection Fraction
IQR	Interquartile Range
KRT	Kidney Replacement Therapy
LVEF	Left Ventricular Ejection Fraction
MRA	Mineralocorticoid Receptor Antagonist
NYHA	New York Heart Association
RAAS	Renin–Angiotensin–Aldosterone System
SBP	Systolic Blood Pressure
SGLT2I	Sodium–Glucose Cotransporter-2 inhibitor
T2DM	Type 2 Diabetes Mellitus
