# Supplementary material for: Cardio-Vasculo-Renal Benefits of SGLT2 Inhibitors in Heart Failure: A Retrospective Study from a Lower-Resource Tertiary Center
Source: Medicina (Kaunas). 2026 Jan 26;62(2):256. doi: 10.3390/medicina62020256 (PMC12942245; doi:10.3390/medicina62020256)
Supplement: Supplementary file 1 [file medicina-62-00256-s001.zip › medicina-4068974-supplementary/medicina-4068974-supplementary.pdf]

Supplementary Table S1. Propensity Score Covariate Balance Before and After Overlap Weighting

Variable	Unweighted SMD	Weighted SMD
Age (years)	0.12	0.03
Male sex	0.02	0.01
Baseline eGFR (mL/min/1.73 m²)	0.07	0.02
CKD (eGFR < 60 mL/min/1.73 m²)	0.01	0.01
Type 2 diabetes mellitus	0.11	0.04
NYHA class III–IV	0.05	0.02
Ischemic HF etiology	0.04	0.01
Atrial fibrillation	0.03	0.01
Systolic blood pressure	0.06	0.02
Anemia	0.03	0.01
RAAS inhibitor / ARNI use	0.02	0.01
Beta-blocker use	0.04	0.02
Mineralocorticoid receptor antagonist use	0.05	0.02
Loop diuretic use	0.03	0.01
Statin use	0.04	0.02

After overlap weighting, all covariates achieved excellent balance with standardized mean differences <0.10, indicating adequate control of measured confounding. Abbreviations: SMD = standardized mean difference; CKD = chronic kidney disease; HF = heart failure; eGFR = estimated glomerular filtration rate; NYHA = New York Heart Association; RAAS = renin–angiotensin–aldosterone system; ARNI = angiotensin receptor–neprilysin inhibitor.

Supplementary Table S2. Safety and Tolerability Outcomes During Follow-Up

Outcome	SGLT2i (n = 110)	non-SGLT2i (n = 130)	p-value
Acute kidney injury (AKI)	9 (8.2%)	13 (10.0%)	0.62
Volume depletion / hypotension	7 (6.4%)	6 (4.6%)	0.53
Genital mycotic infection	6 (5.5%)	1 (0.8%)	0.04
Diabetic ketoacidosis	1 (0.9%)	0 (0.0%)	0.28
Urinary tract infection	10 (9.1%)	12 (9.2%)	0.98
SGLT2i discontinuation due to adverse events	8 (7.3%)	—	—

Values are presented as n (%). Acute kidney injury was defined as a ≥0.3 mg/dL increase in serum creatinine within 48 hours or a ≥1.5-fold increase from baseline. Volume depletion events included symptomatic hypotension or clinical dehydration requiring treatment adjustment. No fatal adverse events related to SGLT2 inhibitor therapy were observed. Overall tolerability was good, with low discontinuation rates.

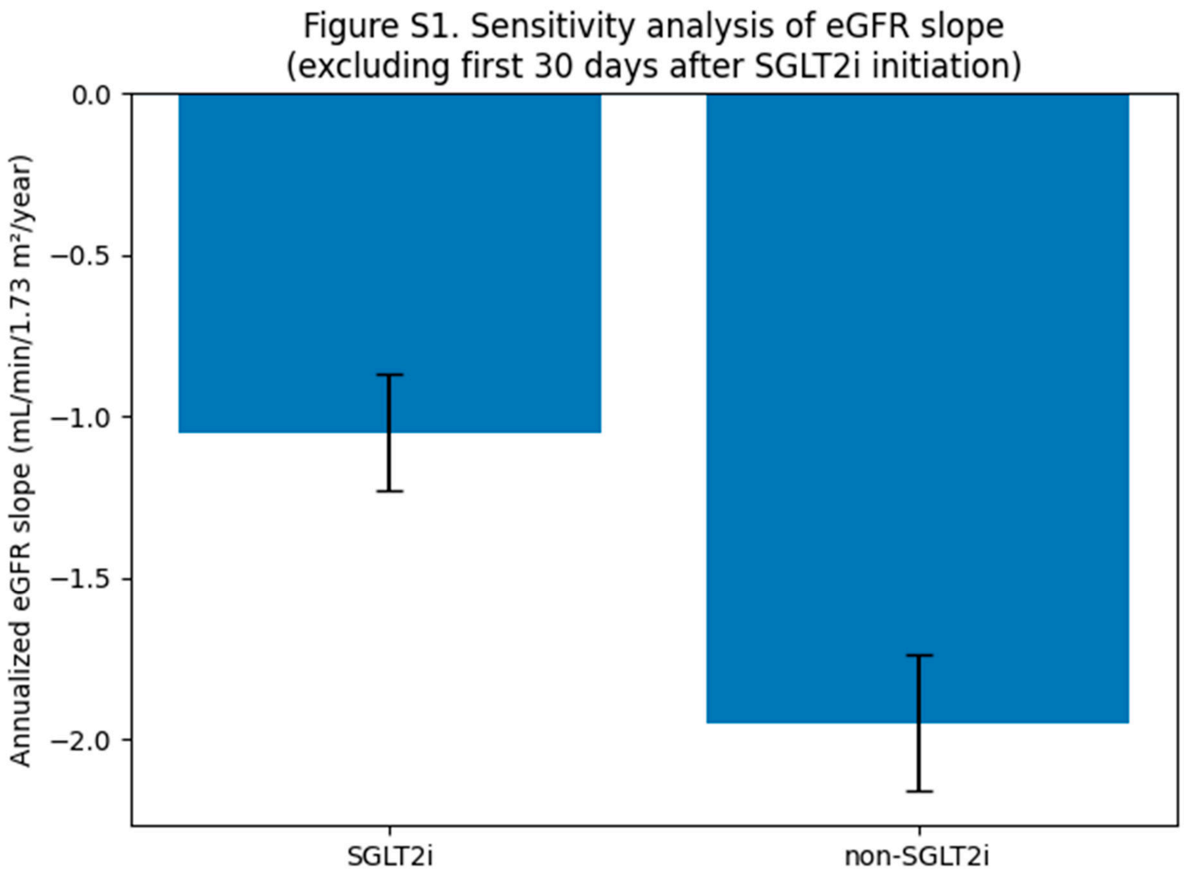

Figure S1. Sensitivity analysis of annualized eGFR slope excluding measurements obtained within the first 30 days after SGLT2 inhibitor initiation.
After exclusion of early post-initiation eGFR values to account for the expected acute hemodynamic dip, patients treated with SGLT2 inhibitors continued to demonstrate a significantly slower rate of kidney function decline compared with untreated patients. Error bars represent standard error of the mean.
